# Case report of neonate Pierre Robin sequence with severe upper airway obstruction who was rescued by finger guide intubation

**DOI:** 10.1186/s12871-019-0754-2

**Published:** 2019-05-22

**Authors:** Li Zhang, Jian Fei, Jian Jia, Xiaohua Shi, Meimin Qu, Hui Wang

**Affiliations:** Department of Anesthesiology, Children’s Hospital of Nanjing Medical University, Nanjing, 210008 Jiangsu Province China

**Keywords:** Pierre Robin sequence, Upper airway obstruction, Finger guide intubation

## Abstract

**Background:**

Pierre Robin Sequence (PRS) patients are known for their triad of micrognathia, glossoptosis, and airway obstruction. Their airway can be a challenge even for the most experienced pediatric anesthesiologist.

**Case presentation:**

We report the case of a 9 day old 3.5 kg boy diagnosed with PRS, cleft palate, and a vallecular cyst with severe upper airway obstruction. The combination of PRS, cleft palate and the presence of vallecular cyst made this a cascade reaction of difficult airway. Due to his unique anatomy, we didn’t appreciate how difficult his airway was until multiple attempts with high-tech equipment failed. Ultimately it was the finger guide intubation, this old technique without any equipment, that rescued this patient from lose of airway.

**Conclusions:**

The boy was successfully rescued by finger guided intubation. Finger guide intubation should be added to the anesthesiologist’s newborn rescue intubation training.

## Background

Pierre Robin Sequence(PRS)patients are known for their triad of micrognathia, glossoptosis, and airway obstruction [1]. In addition to positioning and nasal pharyngeal airway (NPA), newborns with PRS may require surgical treatments including tongue lip adhesion (TLA), mandibular distraction osteogenesis (MDO), subperiosteal release of the floor of the mouth (SPRFM), tracheostomy if their airway obstruction deteriorates or they failure to thrive [2]. To have those procedures done, their airway need to be secured first. Their airway can be a challenge even for the most experienced pediatric anesthesiologist. We describe the case of an anatomical abnormality associated with PRS which complicated attempts at airway management, and the ultimate technique that enabled placement of an endotracheal tube.

## Case presentation

**Fig. 1:**
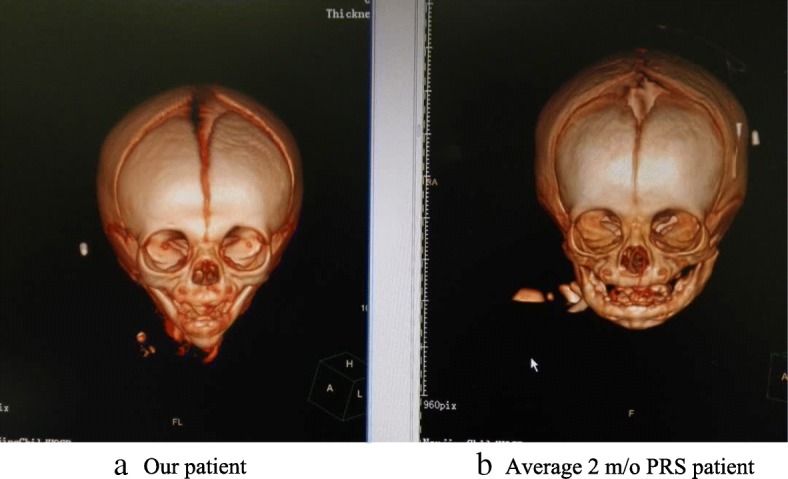
**a** shows the Craniofacial CT reconstruction of our patient. **b** shows the Craniofacial CT reconstruction of a normal 2-months-old PRS patient

A 9 day old 3.5 kg boy was referred to our tertiary care hospital with diagnosed of PRS. Other than atrial septal defect (ASD), aspiration pneumonia and unilateral complete cleft palate with a maximum width of about 0.8 cm. There are no cleft lip or alveolar cleft or any other comorbidity. Upon admission, he presented with cyanosis with venous carbon dioxide pressure (PvCO_2_) 87.8 mmHg, multiple bedside direct laryngoscopy and GlideScope (UE Medical, China) attempts were made however none were successful. His saturation was improved to 95% by facial mask. The next morning he had thin sliced Computed Tomography (CT, Philips) with craniofacial as well as airway reconstruction (Fig. 1a, b).

The same night he deteriorated again. We attempted intubation with GlideScope which revealed grade 4 view. Next we tried a blind intubation with endotracheal tube loaded with stylet, however, this failed as well. Then we tried size 1 laryngeal mask airway (LMA, Well Lead Medical, China), however, we felt the LMA was blocked by an occupying lesion at the left side of tongue’s base so we decided not to force it through for fear it might further aggravate his airway. His respiratory distress was improved after we placed a NPA and saturation returned to 100%.

**Fig. 2:**
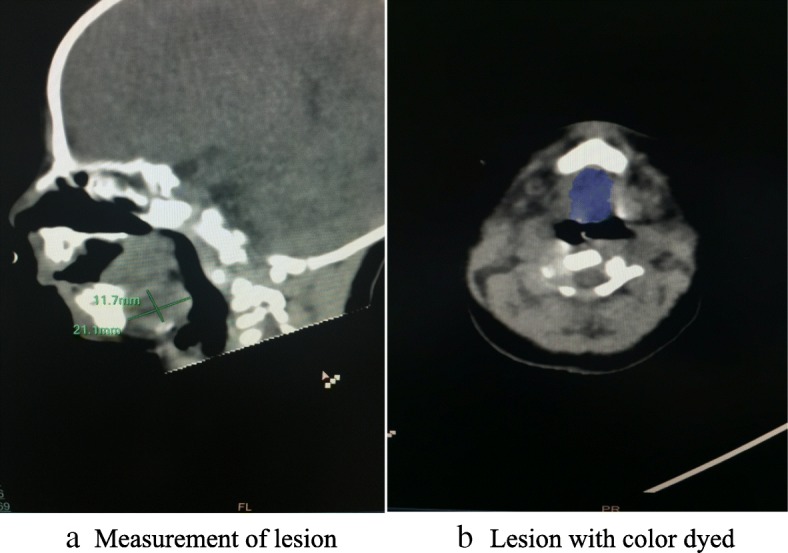
**a** shows the airway CT of our patient. we found he had large lesion with size of 21.1 mm X 11.7 mm occupying his base of tongue extending from left all the way to middle. **b** shows the same lesion with color dyed

The third morning he was brought to operating room for MDO placement. After giving Penehyclidine to dry his secretion, we slowed dialed Sevoflurane to 6% then back to 3% to maintain his spontaneous breathing. Placement of a glidescope revealed no identifiable glottic structures. Fiberoptic scope (Olympus, Japan) revealed the epiglottis lying on the posterior pharynx, which could not be maneuvered beneath. Size 1 LMA and lighted wand (CLARUS Medical, MN) cannot be placed in the right place, multiple attempts with high-tech equipment failed to establish his airway. Since NPA could maintain his saturation, we decided to abort the procedure. Upon arrival in surgery intensive care unit (SICU), his PvCO_2_ was 119.4 mmHg. A TLA procedure was performed with sedation. The fourth night his PvCO_2_ was elevated to 183.8 mmHg. We reviewed his airway CT again with a different radiologist. We found he had large lesion with size of 21.1 mm X 11.7 mm occupying his base of tongue extending from left all the way to middle. Most likely it was thyroglossal cyst per the second radiologist. (Fig. 2a, b).

Knowing his hypercarbia could get even worse, on day 5 we brought him back to the operating room. After inducing patient with ketamine and sevoflurane, operator gloved then advanced nondominant middle finger along the tongue, once patient’s epiglottis was touched, middle finger was bent slightly to lift epiglottis, dominant hand then passed the lubricated and bent endotracheal tube based on 3D reconstruction right next to the middle finger into his trachea. Tube position was confirmed with capnography with endotidal CO_2_ of 120 mmHg. Once airway secured, patient had MDO procedure without any problem. He was sent back to SICU and successfully extubated there on postoperative day 5.

## Discussion

The concept of finger guide intubation was first described in 1543 when Vesalius mentioned how to place a tube into the trachea for control of ventilation. In 1941 Ross and Strong reported using this concept for neonatal resuscitation. Sensing it was not gaining traction among clinicians, in 1968 Woody and Woody advocating this technique again arguing in experienced hands it only took 3–5 s [3]. In 1992 Hancock reported their experiences with finger intubation in newborns and stated it was their preferred method of intubation among physician or nurse once learned [4]. In 2011 Xue pointed out that finger guided intubation in newborns and infants with difficult airways is a possible ignored technique [5].

Nanjing Children’s hospital is one of the largest PRS treatment centers in China. In 2017 alone, we treated 225 patients with PRS including 8 neonates, 24 infants aged 1~3 months, 54 infants aged 3~6 months, as well as 98 infants aged 6~12 months. We are well versed in direct laryngoscopy as well as all advanced airway equipment such as GlideScope, fiberoptic scope, lighted wand, LMA or combination of those instruments. This patient was born by G4P3 mother with 2 normal siblings. We didn’t anticipate too much of difficulty when it was time to secure his airway thinking he was just another patient with PRS. The combination of PRS, cleft palate and the presence of vallecular cyst made this into a cascade reaction of difficult airway. The cyst pushed patient’s epiglottis downward which almost completely obscured the view of patient’s vocal cord. Direct laryngoscopy, glidescope, size 1 LMA, fiberoptic scope as well as lighted wand all failed to establish his airway. Ultimately it was the finger guide intubation, this old technique without any equipment, that eventually rescued this patient from lose of airway. Tracheostomy would have been plan B had digital intubation failed, however, tracheostomy has its own complication such as sudden airway obstruction from accidental decannulation, or mucous plugging; airway infections, tracheal obstruction and inhibition of proper speech and swallowing development.

After this, we made a point to teach this technique to our trainees and junior attending physicians. The contents of the course include guided learning in neonates with normal anatomy/abnormal anatomy and guided learning using manikin models. Familiarity with the technique makes it possible to quickly confirm intubation where unexpected anatomic abnormalities emerges with no immediate availability of hightech airway equipment. Sometimes neonates born with meconium aspiration are hard to be intubated due to poor visualization because of meconium soiling of larynx. Likewise, in ruptured airway vascular abnormality, digital intubation might be the only means to secure patient’s airway when blood gushing out of patient’s mouth. For newborns, the fingers are more flexible than the laryngoscope therefore easier to touch the position of the epiglottis. Plus, there is no need to stoop or bend to adjust eye level, no need for equipment not even lighting source. Having said that, an obvious limitation factor for newborns is the size (airway versus clinician’s finger), it might be very difficult to do digital intubation by a beefy hand trying to negotiate inside a neonate’s very small upper airway.

At a tertiary Children’s Hospital specialized in treating pediatric Pierre Robin Sequence, we had to resort to old fashioned digital intubation to finally secure the airway of this PRS neonate due to unique anatomy. Therefore, perhaps there should be a role of this technique so future anesthesia providers will have one more weapon in their armamentarium of airway management. The anesthesiologist’s newborn rescue intubation training should include the finger guide intubation.

## Abbreviations

ASD: Atrial septal defect
CT: Computed tomography
LMA: Laryngeal mask airway
MDO: Mandibular distraction osteogenesis
NPA: Nasal pharyngeal airway
PRS: Pierre robin sequence
PvCO2: Venous carbon dioxide pressure
SICU: Surgery intensive care unit
SPRFM: Subperiosteal release of the floor of the mouth
TLA: Tongue lip adhesion
